# Deterministic field-free voltage-induced magnetization switching with self-regulated precession for low-power memory

**DOI:** 10.1038/s41598-023-43378-2

**Published:** 2023-09-26

**Authors:** Stanislav Sin, Saeroonter Oh

**Affiliations:** Department of Electrical and Electronic Engineering, Hanyang University, Ansan, 15588 Korea

**Keywords:** Engineering, Electrical and electronic engineering

## Abstract

Spintronic devices are regarded as a promising solution for future computing and memory technologies. They are non-volatile, resilient to radiation, and compatible with the CMOS back-end process. However, the major drawbacks of modern current-driven spintronic devices are the long switching delay and relatively high power consumption. Recent progress in magnetoelectronics, particularly in voltage-controlled magnetism reveals a possible solution. Voltage-controlled magnetic anisotropy (VCMA) allows the manipulation of interface-mediated perpendicular anisotropy energy. However, most VCMA-based switching methods require pre-read operation, precise pulse-width control and have high write error rate. This study proposes a novel deterministic self-regulated precessional ferromagnet switching method, which overcomes these issues. In the discussed method, energy symmetry is broken by a dependence of MTJ resistance on the angle between magnetization vectors of free and pinned layers. Hence, the method does not require an external magnetic field and large electric current. The proposed method is verified through micromagnetic simulations and benchmarked with other methods typically reported in the literature. We report the write error rate is significantly improved compared to other VCMA switching methods. Moreover, the mean energy consumption is as low as 38.22 fJ and the mean switching delay is 3.77 ns.

## Introduction

Density of 2D scaling of conventional transistors and memory devices are either slowing or expanding into the third dimension. Hence, a large number of studies is devoted to technologies which will augment current silicon devices or will be used in emerging computing architectures, such as monolithic integration of logic and memory, compute-in-memory, neuromorphic computing, and probabilistic computing. One of these promising technologies is spintronics^1–7^. Spintronic devices are non-volatile, resilient to radiation, and most importantly, they are compatible with the CMOS back-end process. Spintronic memory can be used as a non-volatile alternative to low-level cache, embedded memory, or a new class of memory^5^. However, there are several challenges to a broad application of spintronics in modern computing, such as long switching delay and large power consumption due to their current-driven operation.

Recently-discovered voltage-controlled magnetic anisotropy (VCMA) effect may become a viable solution to these challenges. Voltage-induced charge at the magnet/oxide interface reduces magnetic anisotropy energy, allowing a lower barrier for magnetization switching^2–5,8–11^. However, VCMA alone cannot perform a 180° switching, and it is used as an assistance to other symmetry breaking-mechanisms. Such a mechanism can be an external magnetic field (ExMF) combined with time-domain asymmetry, spin-transfer torque (STT), or spin-orbit torque (SOT).

Symmetry-breaking by means of ExMF results in a magnetic field-assisted precessional voltage-induced (MFPV) switching method^4,8–14^. In this switching, a short voltage pulse lowers the energy barrier, whereas the ExMF creates energy minima in the plane direction, thus generating a precession. If the pulse timing is right, the precession should finish when magnetization reaches the opposite state. This method results in a sub-nanosecond and low-power magnetization switching, which is attractive. But it also has several drawbacks such as precise pulse timing requirement, non-deterministic bit writing (MTJ can only toggle its state and requires a pre-read operation), and high write error rate (WER)^14–16^. Moreover, current-generated ExMF introduces additional power consumption and leads to cross-talk between adjacent cells. Hence, many efforts are devoted to realize a field-free magnetization switching. Recent studies show that the addition of an antiferromagnetic layer on top of the MTJ can induce an effective in-plane field with a magnitude of $\sim$4000 A/m^17–19^. Additionally, the in-plane term of demagnetizing energy can assist the precession^13,20,21^. Both effects can be used for a field-free precessional voltage-induced (FFPV) switching method. Note that in this work, the term “field-free” implies an absence of ExMF. FFPV eliminates the need for a conducting wire, thus improving cell scalability and simplifying the device design; however, other issues of MFPV remain unsolved. Hence, complex error correction circuitry with self-adjustment and self-termination should be used for precessional switching^22–25^.

It is also possible to divide a voltage pulse into two sub-pulses: the first sub-pulse having enough magnitude to start a precession, whereas a lower second pulse is used to inject a small amount of STT. This method is referred to as precessional STT-assisted (PSTT) switching^4,12,14,26^. PSTT is a field-free and relatively fast switching; however, since it relies on STT, its power efficiency is slightly worse compared to the purely precessional methods. Improper sub-pulse timings theoretically may lead to a false switching, but the timing requirement is not as strict as for MFPV and FFPV.

In-plane field from antiferromagnet can be used for SOT switching of MTJs with perpendicular magnetization^17–19,27–29^. Moreover, external voltage can be applied across the MTJ to utilize barrier lowering from the VCMA effect. This method is referred to as voltage-assisted SOT (VSOT) switching. The advantage of the VSOT method is that it has a separated low-resistivity path for SOT current and a high-resistivity path for voltage. Consequently, pulses used for SOT and VCMA can be injected simultaneously. It allows deterministic, sub-nanosecond switching, but the current density in SOT material is typically on the order of MA/cm$^2$.

Recently, Wu et al. proposed a deterministic and field-free, voltage-induced (DFFV) ferromagnet switching^30^. In their work, energy symmetry was broken by the perpendicular stray field from the synthetic antiferromagnet stack. The magnet is switched from the parallel (P) to the antiparallel (AP) state using a single-pulse voltage, but for AP$\rightarrow$P switching a double-pulse voltage is required. Experimental measurements demonstrated sub-nanosecond delay with 20 fJ energy consumption. However, due to the uncompensated stray field, thermal stability factor $\Delta$ decreases with respect to the nominal value. Hence, a relatively low WER of $\sim$10$^{-4}$ was shown.

In this study, an alternative deterministic field-free switching method is proposed, which utilizes the the fact that $R_{ap}>R_p$ for breaking the energy symmetry. The proposed deterministic field-free self-regulated precessional (DFFSP) method utilizes a double-barrier MTJ configuration, which has high thermal stability and high TMR^31^. Micromagnetic simulations were performed to verify the device operation. We found that the switching delay does not monotonically decrease with current but exhibits oscillating behavior due to the formation of local energy minima along the *x*-axis, revealed by magnetic energy contour maps. Finally, the performance of DFFSP was benchmarked using Monte Carlo simulations and compared with those of other methods.

## Results

### Proposed deterministic field-free magnetization switching with self-regulated precession

**Figure 1:**
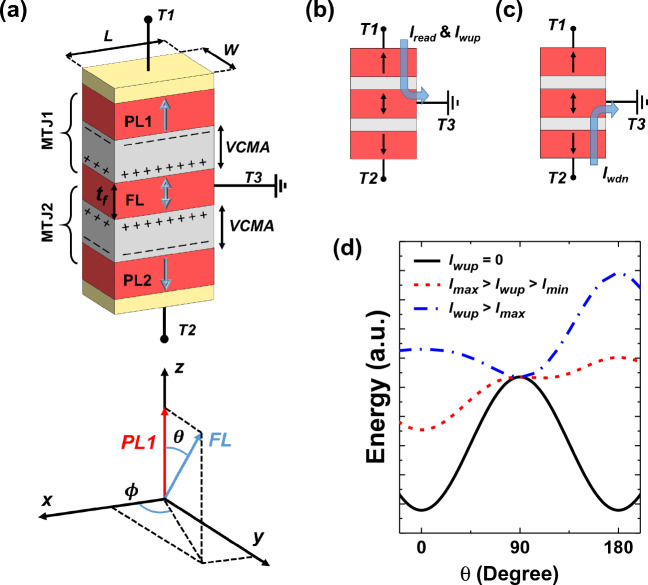
(**a**) Double-barrier MTJ stack for the DFFSP switching and reference coordinate system. (**b**) Writing the “up” state and read operations. The current is injected through terminals T1 and T3. (**c**) Writing the “down” state operation, current is injected through T2 and T3. (**d**) Energy plot for MTJ1 at different magnitudes of $I_{wup}$.

Figure 1a shows the double-barrier MTJ structure for the proposed DFFSP switching scheme. The free layer (FL) is sandwiched between two pinned layers (PLs), which are separated from each other by a tunneling barrier. PL1 (PL in MTJ1) and PL2 (PL in MTJ2) are magnetized in +*z* and -*z* direction, respectively. The device has three terminals: FL is connected to the ground (through terminal T3), whereas PL1 and PL2 are connected to the top (T1) and bottom (T2) terminals, respectively. We assume a generic double-barrier MTJ stack^31–36^: Ta/{[Co/Pt]/Co/Ru/Co/[Co/Pt]} (SAF layer)/Co/Ta/CoFeB (PL1)/MgO/[CoFeB/W/CoFeB] (FL)/MgO/CoFeB (PL2)/Ta/{Co/[Co/Pt] /Co/Ru/Co[Co/Pt]} (SAF layer)/Ta. The double MTJ structure takes advantage of having two oxide/FL interfaces, hence the doubled effective interface anisotropy, compared to the conventional MTJ. A composite CoFeB/W/CoFeb FL layer is used since a thin W spacer further improves interfacial anisotropy of the layer.

The fabrication of such a stack may be difficult since it requires attaching a contact to the FL deposited between PL1 and PL2. We suggest to slightly extend FL out of the main stack. The excessive length of FL can be used for contact formation. In this study we analyze the theoretical upper limit of the DFFSP, thus we assume the extension length of FL is so small that it has almost no effect on the device performance.

As shown in Fig. 1b, writing the “up” state and read operations are performed by feeding a constant current through MTJ1 (connected between T1 and T3). The bias voltage across MTJ1 $V_b$ will induce charge at the top oxide interface, which due to the VCMA effect will decrease perpendicular anisotropy by the amount $E_{VCMA}\propto I_{wup} R(\theta ,V_b)/t_{ox}$, where $I_{wup}$ denotes the current for writing the “up” state, $t_{ox}$ denotes the oxide thickness, and *R* denotes the tunneling resistance which is the function of $V_b$ and angle $\theta$ between the magnetization of PL1 and FL. The energy symmetry is broken due to the VCMA effect since the voltage $I_{wup} \cdot R_p < I_{wup} \cdot R_{ap}$, and if the current is large enough, magnetization will favor the P state. Similarly, MTJ2 allows deterministic writing of the “down” state, by injecting current through terminals T2 and T3 (see Fig. 1c). Figure 1d shows the energy plot for the MTJ1 at different currents. The magnetization reversal is possible for currents $I_{min}<I_{wup}<I_{max}$; however, energy minima is formed in $\pm x$-direction for $I_{wup}>I_{max}$ and switching becomes nondeterministic. Although electric current flows in the device, STT is not utilized. The sole purpose of current is to induce a charge in the oxide layer. Moreover, STT opposes precessional torque and is an unwanted effect in the proposed switching scheme.

### Simulation results

To verify the DFFSP switching, micromagnetic simulations are performed. Table 1 shows FL parameters used in this study, which are based on experimental studies in the literature. As was mentioned before, double-barrier MTJ has almost two times larger interfacial anisotropy constant, which allows using a thicker FL. The advantage of thick FL is the higher in-plane term of demagnetizing field, which helps to switch the device faster.

**Table 1:** Simulation parameters for the free layer.

Symbol	Value	Description	Ref.
$M_s$	$6.25\times 10^5$ A/m	Saturation magnetization	^10^
$\Delta$	60.0	Thermal stability factor	^10^
$K_i$	0.68 mJ/m$^{2\;*}$	Interfacial anisotropy constant	^10^
$\xi$	100 fJ/V$\cdot$m	VCMA coefficient	^37,38^
$\alpha$	0.05	Damping constant	^4,16^
*P*	0.58	Spin polarization	^10^
$L\times W$	$110\times 50$ nm$^2$	Area of MTJ	
$t_f$	3.0 nm	Thickness of th FL	
$t_{ox}$	1.65 nm	Oxide thickness	
$RA_p$	758.7 $\Omega \cdot \mu$m$^2$	Resistance-area product	^39,40^
*TMR*	250%	Tunneling magnetoresistance	^39,40^
$V_h$	0.65 V	Voltage at which $R_{ap}$ halves	^39,40^

*Note that interface anisotropy is doubled for a double-barrier MTJs^31–36^.

**Figure 2:**
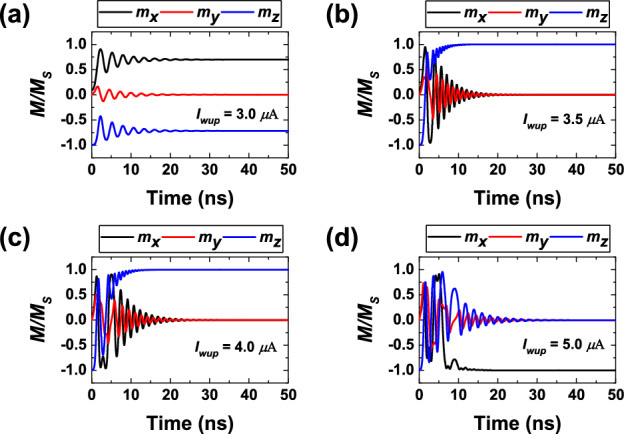
Temporal evolution of the normalized magnetization ($\varvec{M}/M_s$) at $I_{wup}$ of (**a**) 3 $\mu$A, (**b**) 3.5 $\mu$A, (**c**) 4 $\mu$A, and (**d**) 5 $\mu$A. Note how the current magnitude affects $m_z$ switching.

Figure 2a–d show the magnetization dynamics under the influence of a current pulse with a duration of 50 ns. Here, the effect of random thermal field was excluded for clarity. Figure 2a shows that 3 $\mu$A current is insufficient for switching, therefore, such current can be used for the read operation. It induces voltage of 0.81 V and $m_z$ establishes at -0.75. The current of 3.5 $\mu$A allows magnetization switching in approximately 4.6 ns. During the switching, voltage changes from 0.90 V in AP state to 0.48 V in P state. Magnetization switching also occurs at 4 $\mu$A, where voltage across the MTJ varies from 0.97 V to 0.55 V. However, magnetization oscillates back and forth before achieving the parallel state, which results in the switching delay to increase to $\approx$ 7 ns. The current of 5 $\mu$A induces 1.12 V. It leads to precession, and therefore, deterministic switching is not possible anymore. Such current dependence can be explained by means of energy contour maps.

**Figure 3:**
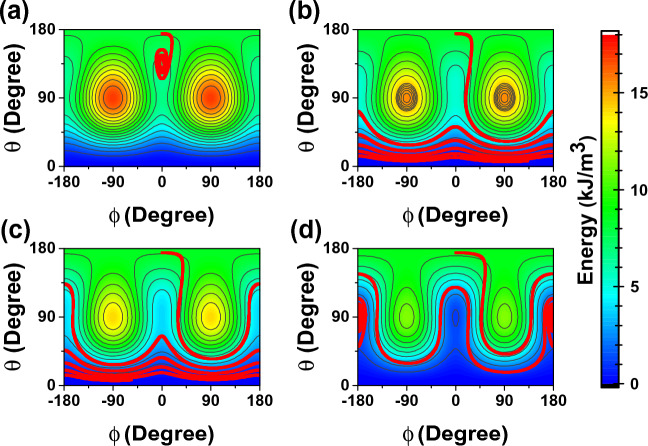
Magnetization trajectory (thick solid line) mapped on the energy contour plots at $I_{wup}$ of (**a**) 3 $\mu$A, (**b**) 3.5 $\mu$A, (**c**) 4 $\mu$A, and (**d**) 5 $\mu$A. Higher current leads to the formation of energy minima at $\pm x$-direction.

The magnetization trajectories mapped on the energy contour plots in $\theta \phi$-plane are shown in Fig. 3a–d. As shown in Fig. 3a, at 3 $\mu$A a small energy barrier between AP and P states still exists and prevents switching. As shown in Fig. 3b, the barrier is eliminated at 3.5 $\mu$A, there is now an energy minimum at $\theta =0^{\circ }$, and switching is possible. It is also the case for 4 $\mu$A, but thanks to the demagnetizing energy, the local energy “pockets” are formed near the *x*-axis, as can be seen in Fig. 3c. The magnetization dwells into these pockets, thus increasing the switching delay. Eventually, at the current of 5 $\mu$A, these “pockets” deepen, and the magnetization vector is trapped in them (see Fig. 3d). Thus, to prevent the formation of “pockets” an appropriate magnitude of current should be used. The study shows that current margin can be increased by decreasing the aspect ratio (AR). However, the switching delay will be degraded.

### Analysis of performance sensitivity to material parameters

**Figure 4:**
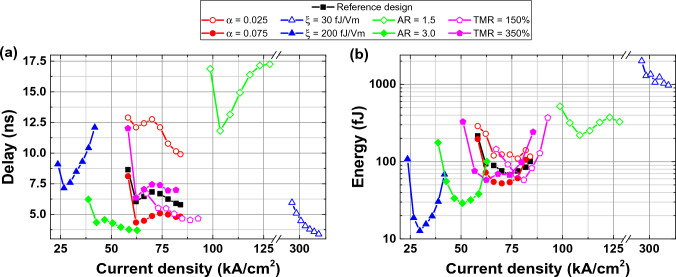
(**a**) Delay versus current density characteristics (in linear scale) and (**b**) energy versus current density characteristics (in logarithmic scale) sensitivity to changing material parameters with respect to the baseline design. Baseline design has $\alpha =0.05$, $\xi =100$ fJ/V$\cdot$m, AR = 2.2, TMR = 250% is shown as rectangles.

Delay versus current density and energy consumption versus current density plots for a baseline design are shown in Fig. 4a and b, respectively. The current was swept from a lower critical switching value of 54.54 kA/cm$^2$ to the maximum value of 90.91 kA/cm$^2$, since higher current densities lead to the loss of deterministic switching. As can be seen, the switching delay does not monotonically increase with the current. An optimal point with 6.25 ns and 75.91 fJ was observed at 77.82 kA/cm$^2$. At higher currents, switching energy rises significantly, whereas delay decreases negligibly.

Next, the effect of the damping constant $\alpha$ was studied. Generally, a higher damping constant is preferred because it improves both delay and energy consumption. A high $\alpha$ can be achieved by decreasing FL thickness or choosing optimal annealing temperature during stack formation^41,42^. For $\alpha =0.075$, optimal delay decreases to 4.87 ns with 52.51 fJ energy, whereas for $\alpha =0.025$, they are 9.91 ns and 116.52 fJ, respectively.

VCMA coefficient $\xi$ has a notable effect on the delay. For $\xi =30$ fJ/V$\cdot$m, delay changes in the range of 5.96–3.39 ns, whereas, for $\xi =200$ fJ/V$\cdot$m, its range is 12.09–8.48 ns. Such behavior can be explained by a steeper energy density landscape at high $\xi$. When the energy landscape is steep, magnetization gets less acceleration from the effective in-plane demagnetizing field; therefore, the switching delay is higher. The major effect of $\xi$ is the change in operating current margin, which leads to a decrease in energy. The current is on the order of hundreds of kA/cm$^2$ for 30 fJ/V$\cdot$m and tens of kA/cm$^2$ for 200 fJ/V$\cdot$m. It resulted in a drastic change of energy from 975.24 to 12.71 fJ. Note that $\xi >200$ fJ/V$\cdot$m is theoretically predicted for FePt/MgO interface^20,21^, and $\xi >300$ fJ/V$\cdot$m was experimentally demonstrated for Fe/MgO^38^.

The effect of changing the AR was studied by fixing the width to $W=50$ nm while changing the length of the MTJ. As can be seen in Fig. 4a and b, AR = 1.5 results in considerable performance degradation: delay is on the order of 10 ns, and energy is $\sim$200 fJ. AR = 3 improves both delay and energy consumption compared to the baseline design. They are as low as 3.77 ns and 38.22 fJ, respectively. It can be concluded that even though shape anisotropy is not necessary for the switching, its effect can be significant. However, the maximum AR of the MTJ may also be limited by manufacturing reasons.

Decreasing the TMR ratio leads to a minor delay improvement. It also can be explained by decreased energy slope for lower TMR. At TMR = 150% optimal point is 5.05 ns with 58.04 fJ, which is achieved at 80.91 kA/cm$^2$. If TMR is increased to 350%, the optimum becomes 7.04 ns with 57.90 fJ at 62.36 kA/cm$^2$. Note that energy consumption was reduced compared to the baseline design.

Although high $\alpha$ and $\xi$ are desired for the DFFSP switching, they are process-defined. Consequently, their optimization involves considerable effort. In contrast, AR can be easily changed to improve cell performance. However, if the width of the MTJ equals minimal feature length F, AR can only be increased with the cell area.

### DFFSP performance benchmarking

DFFSP was benchmarked to other switching methods frequently reported in the literature, such as STT, MFPV, FFPV, and PSTT. For a fair comparison, the material parameters from Table 1 were kept constant and only the stack geometry was optimized. Experimental results for VSOT^19,28^ and DFFV^30^ were included for the sake of comparison.

STT magnetization reversal is purely current-induced and favors low-resistivity junctions^5,6^. As can also be seen from Eq. (3), STT is inversely proportional to the FL thickness. Therefore, oxide and FL thicknesses were reduced to 1.1 nm. At such $t_{ox}$, $RA_{p}=$ 32.98 $\Omega \cdot \mu m^2$, TMR = 75%^39,40^. MTJ area was 50 $\times$ 50 nm$^2$. STT performance was evaluated at different current densities, below the barrier breakdown threshold.

**Figure 5:**
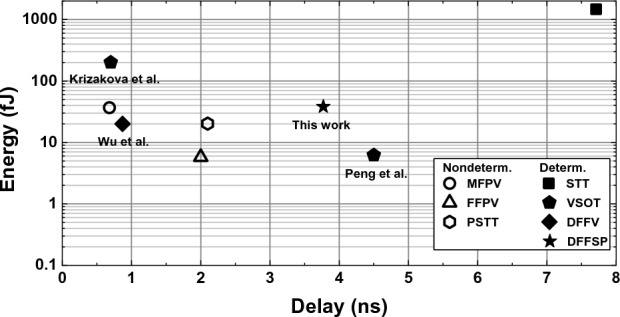
Comparison of switching delay and corresponding switching energy for different switching methods.

**Table 2:** Performance benchmarking and requirements among switching methods.

	STT	MFPV	FFPV	PSTT	VSOT	DFFV^30^	DFFSP (This work)
Delay (ns)	7.74	0.68	2.0	2.1	4.5^19^, 0.7^28^	0.87	3.77
Energy (fJ)	1470	36.8	6.22	22.31	6.2^19^, $\sim$200^28^	20	38.22
Current density (MA/cm$^2$)	4.6	0.09 (MTJ) 437 (wire)	0.09	0.33	5^19^, 170^28^	–	0.06
Pre-read	Not needed	Essential	Essential	Essential	Not needed	Not needed	Not needed
Pulse timing control	Not needed	Essential	Essential	Two pulses (consecutive)	Two pulses (simultaneous)	Two pulses (consecutive)	Not needed
ExMF (kA/m)	No	20	No$$^*$$	No$$^*$$	No$$^*$$	No$$^{**}$$	No
WER	$<10^{-9}$	$10^{-6}$^[,14–16^	$4.5\cdot 10^{-3}$	$<10^{-4}$	–	$\sim 10^{-4}$	$<10^{-9}$

*Small bias field $H_x=$ 4 kA/m provided from the antiferromagnetic stack to assist switching^17–19^.

**Small stray field $H_z=$ 4 kA/m provided from the permanent magnet^30^.

For MFPV and FFPV, $t_f$ was set to 1.1 nm, because $E_{VCMA}\propto 1/t_f$. For MFPV benchmarking, in-plane ExMF $H_x=$ 20 kA/m was assumed to be generated from the current-carrying copper word line. Cell spacing of $2W=$ 100 nm and wire cross-section of 160$\times$10 nm$^2$ was considered. According to our simulations, a current of $\approx$ 7 mA is required to generate such a field. The power associated with one cell was 51.45 $\mu$W. In case of FFPV, $H_x=$ 4 kA/m was assumed to originate from the antiferromagnet.

The PSTT method uses two sub-pulses: a high-voltage sub-pulse to utilize VCMA and a low-voltage sub-pulse to utilize STT. It requires the injection of non-negligible current during the second sub-pulse. Hence, $t_{ox}=$ 1.4 nm with $RA_p=$ 186.11 $\Omega \cdot \mu m^2$ was used. Delay and energy consumption were evaluated for different voltage magnitudes of both sub-pulses. The duration of the first sub-pulse was chosen to avoid false switching. ExMF was excluded from the simulations to study the field-free performance of this method.

Figure 5 shows the results of benchmarking. STT switching is the most energy-demanding with a mean delay of 7.74 ns and mean writing energy of 1470 fJ. The MFPV has a delay of 0.68 ns, which is the fastest among the compared methods. However, the energy is 36.80 fJ. Note that energy dissipated on the MTJ ($\sim$1.8 fJ) is only a small share of the total energy. FFPV method with a 2.0 ns delay is a few times slower, compared to its field-assisted counterpart, but its advantage is the low energy consumption of 6.22 fJ. The PSTT method has a delay of 2.10 ns and energy of 22.31 fJ. For VSOT method, two studies were considered. In the study of Peng et al.^19^ delay of $\sim$4.5 ns with 6.2 fJ energy was shown. Krizakova et al.^28^ reported sub-nanosecond delay of 0.7 ns, but they did not mentioned switching energy. We approximately estimate energy losses on the order of 200 fJ, considering relatively large current densities. Wu et al. demonstrated DFFV MTJ switching with 0.87 ns delay and 20 fJ. The proposed DFFSP method is slower than traditional field-free methods with a delay of 3.77 ns and energy of 38.22 fJ. However, we emphasize that a pre-read-free, single-pulse operation may compensate for the difference in delay and energy. Note that a flat comparison in terms of the delay and energy may give an incomplete picture. A more detailed comparison of the switching methods is given in Table 2.

## Discussion

In summary, we proposed a DFFSP magnet switching method. Unlike the other switching methods, DFFSP utilizes angle dependence of MTJ resistance in order to create energy asymmetry under a constant current. This energy asymmetry favors the parallel state. Therefore, deterministic switching was achieved by the implementation of a double-barrier MTJ structure, which has a benificial interface anisotropy constant and large TMR. DFFSP method demonstrates mean switching delay of 3.77 ns with energy consumption of 38.22 fJ. Furthermore, it combines robust deterministic writing, control simplicity, and energy efficiency to be used in low-power memory applications with low WER.

Even though DFFSP does not have a lower delay compared to other VCMA switching methods, it does not require a pre-read operation nor precise pulse timing, which would reduce WER significantly and dependence on error correction circuitry. Along with low operating current density, DFFSP switching double-barrier MTJ device is a viable candidate for deterministic, high precision, low-power memory applications.

## Methods

The motion of the magnetization vector $\vec {m}$ was modeled by the Landau–Lifshitz–Gilbert (LLG) equation:

1 $$\begin{aligned} \frac{d\vec {m}}{dt}=-\frac{\gamma _0}{(1+\alpha ^2)} \biggl [ \vec {m}\times \vec {H}_{eff} -\frac{\alpha }{\gamma _0}\vec {m}\times \frac{d\vec {m}}{dt} -\frac{P I_s \hbar }{2q\mu _0 M_s V_f}\vec {m}\times (\vec {m}\times \vec {m}_p) \biggr ] \end{aligned}$$

where $\gamma _0$ is the gyromagnetic ratio; $\alpha$ is the damping constant; $\vec {H}_{eff}$ is the effective field; and the last term represents damping STT. Note that due to the low current, STT is significantly lower than the field torque, and its effect can be neglected. Here it was introduced for the sake of completeness. The remaining notations are as follows: *P* is the spin polarization; $\hbar$ is the reduced Planck’s constant; *q* is the elementary charge; $\mu _0$ is the vacuum permittivity; $M_s$ is the saturation magnetization; $V_f$ is the volume of free layer; $\vec {m}_p$ is the PL’s magnetization. $\vec {H}_{eff}$ can be expressed in terms of the free energy density as^4,5,13,43^:

2 $$\begin{aligned} \vec {H}_{eff}&=-\frac{1}{\mu _0M_s}\frac{dE_{tot}}{d\vec {m}}+\vec {H}_{th} \end{aligned}$$

3 $$\begin{aligned} E_{tot}&=-\frac{K_i}{t_f}m_z^2 +\frac{\xi I_s R(\theta ,V_b)}{t_ft_{ox}}m_z^2+\frac{M_s^2\mu _0}{2} \times (N_xm_x^2+N_ym_y^2+N_zm_z^2) \end{aligned}$$

where $E_{tot}$ is the total free energy density, which consists of anisotropy, VCMA, and demagnetization energy terms. $K_i$ is the interface anisotropy constant; $t_f$ is the FL thickness; $t_{ox}$ is the oxide thickness; $\xi$ is the VCMA coefficient; $N_{x}$, $N_{y}$, $N_{z}$ are the demagnetizing factors. Random field $\vec {H}_{th}$ models the Brownian motion of the magnetization and is given as follows:

4 $$\begin{aligned} \vec {H}_{th}=\vec {\sigma }\sqrt{2k_B T \alpha /(\gamma _0 \mu _0 M_s V_f\Delta t)} \end{aligned}$$

where $k_B$ is the Boltzmann constant; *T* is the temperature; $\Delta t$ time discretization step; and $\vec {\sigma }$ is the vector whose elements are the Gaussian distributed random numbers.

The thermal stability factor was calculated by the following analytical formula:

5 $$\begin{aligned} \Delta =\frac{E_b}{k_B T}=\frac{(K_i/t_f-0.5 M_s^2[N_z-N_y])\cdot {}V_f}{k_B T} \end{aligned}$$

Resistance is modeled as the following equation^4^:

6 $$\begin{aligned} R(\theta ,V_b)=R_p\frac{1+(V_b/V_h)^2+TMR}{1+(V_b/V_h)^2+TMR[1/2\cdot (1+cos\theta )]} \end{aligned}$$

where $R_p$ is the resistance in parallel state and $V_h$ is the voltage at which $R_{ap}$ halves. The equation is solved self-consistently.

In this study, LLG was used within the single-domain approximation. Typically, such a model is used for describing small enough magnetic structures where the demagnetizing energy is insufficient to divide the material into multiple domains. It cannot account for a few second-order effects; however, it is still viable in illustrating physics-based device operation.

Spintronic devices are susceptible to thermal fluctuations; therefore, switching delay has stochastical distribution. To evaluate this distribution, Monte Carlo simulations with varying thermal field and initial angle $\theta _0$ were performed. The number of attempts was $n_{at}=$ 10 000. Distribution of the $\theta _0$ was obtained by device simulation at $V_{b}=0$ V. We found that $\theta _0$ varied from 152.07° to 180° with a mean value of 173.30°. The $\theta _0$ was sampled over a 1 $\mu$s period with a sampling frequency of $f_s$ = 4 THz. This distribution was used to generate the initial position for each attempt.

To study better ways of performance optimization, material sensitivity analysis was performed. Parameters listed in Table 1 were chosen as a baseline. Damping constant $\alpha$, VCMA coefficient $\xi$, TMR, and AR were varied one by one while the remaining parameters were fixed. The only exception is the interface anisotropy $K_i$, which was changed to keep thermal stability factor $\Delta$ constant. Switching delay and energy distributions were obtained by Monte Carlo simulations considering the random thermal fluctuations. The switching threshold was set as $m_z=0.9$.

## Data Availability

The data that support the findings of this study are available from the corresponding author upon reasonable request.
